# Anaerobic 3-methylhopanoid production by an acidophilic photosynthetic purple bacterium

**DOI:** 10.1007/s00203-021-02561-7

**Published:** 2021-09-16

**Authors:** Marisa H. Mayer, Mary N. Parenteau, Megan L. Kempher, Michael T. Madigan, Linda L. Jahnke, Paula V. Welander

**Affiliations:** 1 Department of Earth System Science, Stanford University, Stanford, CA 94305 USA; 2 Exobiology Branch, NASA Ames Research Center, Moffett Field, CA 94035 USA; 3 Department of Microbiology and Plant Biology, University of Oklahoma, Norman, OK 73019 USA; 4 Department of Microbiology, Southern Illinois University, Carbondale, IL 62901 USA

**Keywords:** Anoxygenic phototrophs, *Rhodopila globiformis*, Hopanoids, Warm thermal springs

## Abstract

Bacterial lipids are well-preserved in ancient rocks and certain ones have been used as indicators of specific bacterial metabolisms or environmental conditions existing at the time of rock deposition. Here we show that an anaerobic bacterium produces 3-methylhopanoids, pentacyclic lipids previously detected only in aerobic bacteria and widely used as biomarkers for methane-oxidizing bacteria. Both *Rhodopila globiformis*, a phototrophic purple nonsulfur bacterium isolated from an acidic warm spring in Yellowstone, and a newly isolated *Rhodopila* species from a geochemically similar spring in Lassen Volcanic National Park (USA), synthesized 3-methylhopanoids and a suite of related hopanoids and contained the genes encoding the necessary biosynthetic enzymes. Our results show that 3-methylhopanoids can be produced under anoxic conditions and challenges the use of 3-methylhopanoids as biomarkers of oxic conditions in ancient rocks and as prima facie evidence that methanotrophic bacteria were active when the rocks were deposited.

## Introduction

Hopanoids are triterpenoid lipids that support membrane integrity and permeability in certain bacteria (Ricci et al. 2017). Hopanoids are also quite recalcitrant biomolecules; their hopane derivatives can be preserved in sedimentary rocks for billions of years, and because of this, have been exploited as biomarkers of past environmental conditions or particular microbial activities (Brocks et al. 2005). While hopanoids are produced by metabolically diverse bacteria, hopanoids methylated in the A-ring are more restricted in their distribution and linked to particular bacterial taxa or aerobic metabolisms. For example, hopanoids methylated at the C-2 position (2-methylhopanoids) have traditionally been linked to cyanobacteria (Summons et al. 1999) whereas hopanoids methylated at the C-3 position (3-methylhopanoids) have been associated with strictly aerobic methane-oxidizing and acetic acid-oxidizing bacteria (Zundel and Rohmer 1985).

In 2007, 2-methylhopanoid production was reported in photosynthetically grown cultures of the purple nonsulfur (PNS) bacterium *Rhodopseudomonas* (*Rps*.) *palustris* (Rashby et al. 2007), a widely distributed species that inhabits freshwater lakes and fertile soils (Harwood and Gibson 1988). PNS bacteria are a phylogenetically diverse group of anoxygenic phototrophs that preceded cyanobacteria on Earth by at least 500 million years and whose photosynthetic metabolism is strictly anaerobic (Hohmann-Marriott and Blankenship 2011). These *Alpha-* and *Betaproteobacteria* inhabit various aquatic environments, including lakes, wastewaters, hot springs, and marine and hypersaline waters and typically can conserve energy from both photosynthesis (anoxic/light) or respiration (oxic/dark) (Madigan and Jung 2009). Subsequent metagenomic analyses showed that *hpnP*, the gene encoding the enzyme that methylates hopanoids at the C-2 position, was present in microbes from samples of a variety of microbial ecosystems, suggesting that 2-methylhopanoid production is widespread in nature (Ricci et al. 2015). Collectively, these discoveries demonstrated the danger in unambiguously linking the presence of 2-methylhopanes (the breakdown product of 2-methylhopanoids) in ancient sediments to cyanobacteria or to oxic conditions in general.

**Fig. 1:**
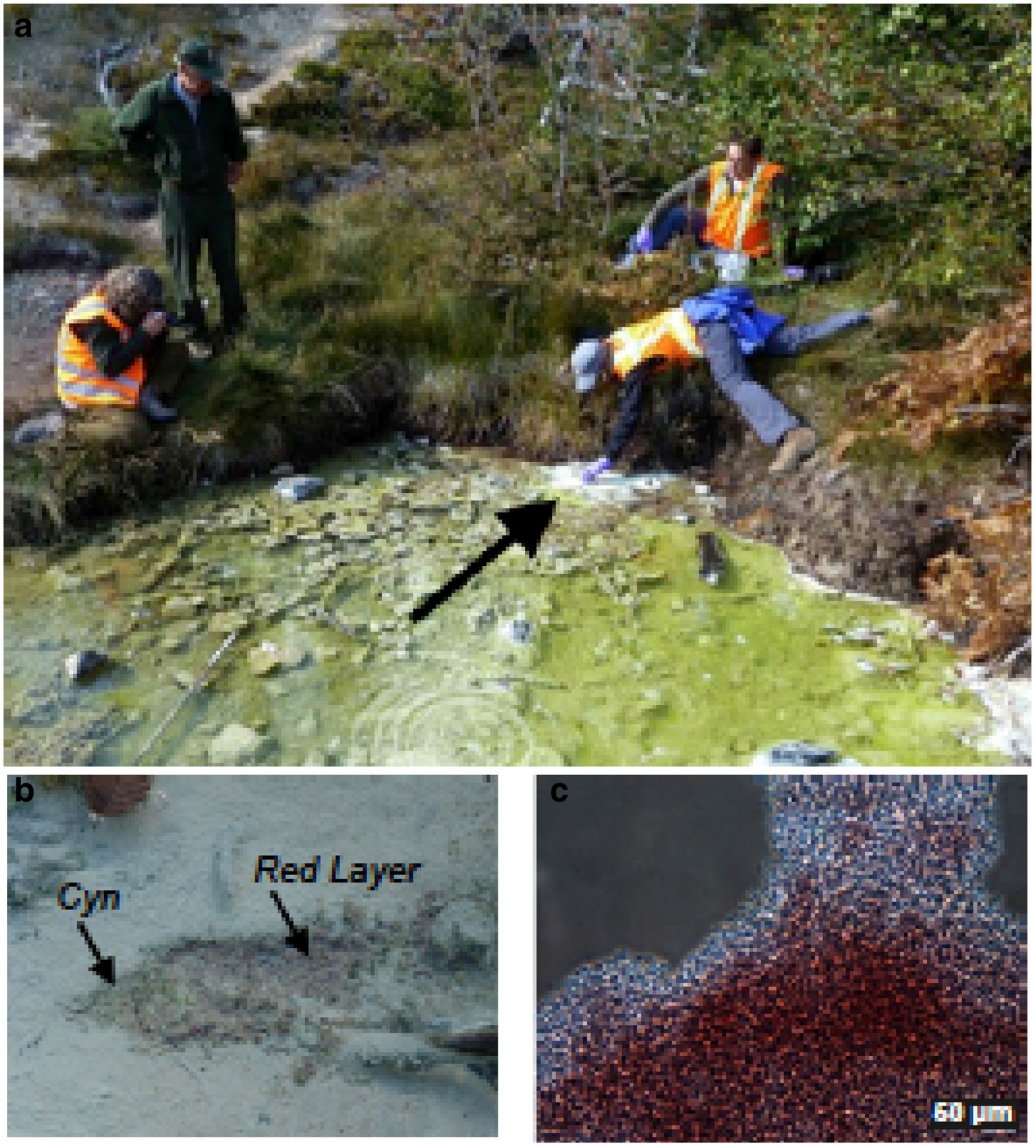
**a** Acidic (pH 3.9) and sulfidic warm spring in Lassen Volcanic National Park. **b** Sampling area designated by black arrow in **a**. The microbial mat contains the red alga *Cyanidium* (*Cyn*) overlying a red layer from which *Rhodopila* strain LVNP was isolated; the white area is elemental sulfur. **c** Phase-contrast photomicrograph of a dense suspension of cells of strain LVNP

In contrast to 2-methylhopanoids, evidence to date has shown that hopanoids methylated at the C-3 position (3-methylhopanoids) (Summons et al. 1999; Rashby et al. 2007) are synthesized only by bacteria that perform O_2_-dependent metabolisms, with the most prominent producers being aerobic methane-oxidizing bacteria (methanotrophs) and acetic acid bacteria (Zundel and Rohmer 1985). The *hpnR* gene that encodes the C-3 hopanoid methylase has also been detected in a diverse array of aerobic bacteria further confirming the link to O_2_-dependent metabolisms (Welander and Summons 2012). Moreover, lipid analyses of microbial mat samples from hypersaline environments detected 3-methylhopanoids in various bacteria, including species of PNS bacteria (Jahnke et al. 2014). In our studies of hot spring microbial mats, we detected 3-methylhopanoids in mat samples collected from a sulfidic and acidic (pH 3–4) spring in Lassen Volcanic National Park (California, USA) that was fed by a continuous discharge of warm volcanic water containing CO_2_, H_2_, and H_2_S (Fig. 1a, b). The mat lacked cyanobacteria but contained a purple-red layer underneath a green-pigmented algal layer. Knowing that 3-methylhopanoids have not been reported from algae, we pursued the Lassen purple bacterium as the possible source of these lipids and, using standard enrichment and isolation techniques, obtained a pure culture of this phototroph we have designated *Rhodopila* strain LVNP, most closely related to another acid spring dwelling PNS, *Rhodopila globiformis* (Fig. 1c). Here we show that pure cultures of this organism and its phylogenetic close relative produce a suite of 3-methylhopanoids when grown under strictly anoxic conditions—the first report of the production of these hopanoid lipids in an anaerobically-grown bacterium—and that their genomes encode the requisite enzymes for methylating these lipids in the C-3 position.

## Materials and methods

### Organisms, isolation, and growth conditions

Lipid analyses and genomic studies were performed on axenic cultures of four purple nonsulfur bacteria: *Rhodopila* strain LVNP, *Rhodopila globiformis* 7950 isolated from Yellowstone National Park (YNP), *Rhodoblastus acidophilus* 7050, and *Rhodopseudomonas palustris* DSM127 (the latter three species from the collection of MTM) (Table 1). *Rhodopila* strain LVNP was isolated from a microbial mat that formed in an acidic (pH 3.9), sulfidic spring in Lassen Volcanic National Park (near 40° 27′ 09.5″ N 121° 32′ 13.3″ W, Northern California, U.S.A.). The mat had an upper algal layer and a lower purple–red layer (Fig. 1). A sample of the purple layer was incubated in liquid medium (Pfennig 1974) under anaerobic photosynthetic conditions at 25 °C, and an axenic culture was eventually obtained from successive transfers of isolated colonies grown on plates of the same medium incubated in a Mitsubishi AnaeroPack 2.5L Rectangular Jar (Thermo Scientific Cat No. R685025). Anoxic conditions in liquid culture media were achieved by first vigorously sparging under filtered argon (0.2 µm filter) for 5 min, and then reducing the media with a sodium sulfide solution to scavenge any remaining oxygen. The 100 mM sodium sulfide stock solution was anoxically prepared, neutralized to pH 7.5, aseptically filtered, and added to the sparged media to a final concentration of 0.14 mM prior to inoculation. To further ensure anoxia during incubation, inoculated anoxic media were transferred to tissue culture flasks and incubated in the AnaeroPack Jar under incandescent light for anaerobic photosynthetic growth. Anoxic conditions are important to maintain carefully for photosynthetic growth of *Rpi. globiformis* and other classical anoxygenic phototrophs because oxygen represses pigment synthesis in these organisms (Yildiz et al. 1991). Our cultures were also grown aerobically under darkness to compare the lipid composition of aerobic and anaerobic growth (Table 1). Our dark, aerobic cultures were grown in the same liquid media and tissue culture flasks as our photosynthetic, anaerobic cultures; however no sparging, no addition of sulfide, and no incubation in the AnaeroPack Jar were applied to the media. The *Rpi. globiformis* 7950 YNP (DSM161), *Rbl. acidophilus* 7050, and *Rps. palustris* DSM127 were grown under similar anaerobic photosynthetic conditions at 25 °C.

**Table 1:** Hopanoid and other pentacyclic triterpenoid composition of pure cultures analyzed in this study via GC-MSD

Organism	Growth conditions	Diploptene (μg/g)	Diplopterol (μg/g)	Tetrahymenol (μg/g)	Bacteriohopane-polyols (μg/g)	3-methylhopanoids (μg/g)	3-methylhopanoid/ Total hopanoids
*Rhodopila* strain LVNP	Anaerobic photohetero-trophic growth in light	3.8	nd	nd	26	3.7	0.14
*Rhodopila* strain LVNP	Aerobic growth in dark	37	nd	nd	40	1.7	0.04
*Rhodopila globiformis* 7950 YNP	Anaerobic photohetero-trophic growth in light	43	nd	nd	123	4.7	0.04
*Rhodoblastus acidophilus* 7050^a^	Anaerobic photohetero-trophic growth in light	270	nd	nd	122	nd	nd
*Rhodopseudomonas palustris* DSM127^b^	Anaerobic photohetero-trophic growth in light	101	37	253	140	nd	nd

*LVNP* Lassen Volcanic National Park, *YNP* Yellowstone National Park, *3-methylhopanoid* 3-methylbacteriohopanepolyol, *nd* not detected

^a^*Rhodoblastus acidophilus* 7050 is the same culture as analyzed by Rohmer et al. (1984) for hopanoids

^b^*Rhodopseudomonas palustris* DSM127 additionally synthesized 49.2 μg/g 2-MeDiploptene, 37.3 μg/g 2-MeDiplopterol, 135.7 μg/g 2-MeTetrahymanol, 4.2 μg/g 2-methylhopanoid, and displayed a ratio of 0.03 2-methylhopanoid/Total hopanoid

### Lipid and genomic analyses

For analysis of lipids, cells were harvested from cultures by centrifugation at 4500 × *g* for 10 min at 4 °C, frozen at − 80 °C, then freeze-dried for storage prior to analysis. The freeze-dried cells were resuspended in water and extracted using a modified Bligh–Dyer (B–D) protocol as previously described (Jahnke et al. 1992). Briefly, the initial B–D solvent phase of cell/water–methanol–chloroform (4:10:5) was sonicated for one hour, then physically agitated by vigorous shaking. The solvent phase (lipid/water–methanol–chloroform) was removed after centrifuging to pellet cellular residue and the extraction procedure repeated. The solvent phase of each extraction was separated by addition of chloroform and water to a final ratio of 9:10:10, the resulting bottom chloroform layers containing the lipid was removed and pooled to generate a total lipid extract (TLE).

A portion of the TLE was analyzed for intact polar lipid fatty acids (IPFA) by alkaline methanolysis (Jahnke et al. 2001). Another portion of the TLE was treated with the oxidation–reduction procedure as described by Rohmer et al. (1984), which removes the polyol side chain from the hopanoid molecule. The resulting extended hopanol products and free hopanoids (diploptene, diplopterol) were derivatized with acetic anhydride (Rohmer et al. 1984) and were analyzed using an Agilent 5977A Gas Chromatograph–Mass Selective Detector (GC–MSD) equipped with a 60 m DB5ms fused silica column. For quantitation, cholestanol-acetate and dibehenoyl-phosphatidylcholine were used as a hopanol and IPFA internal standards, respectively. The quantified hopanoids and other pentacyclic triterpenoids were normalized by grams of lyophilized biomass extracted to allow comparison between cultures (Table 1). The recovered hopanol, 2-methyl- and 3-methylhopanol products were identified based on their mass spectra and retention times as described in Summons and Jahnke (1990) and references therein, and elution patterns reported in Sessions et al. (2013). Briefly, the mass fragment 191 is diagnostic of unmethylated hopanoids, while the 205 fragment is diagnostic of their methylated equivalent. Also, 3-methylhopanoids elute after their unmethylated equivalents, whereas, 2-methylhopanoids elute before.

For liquid chromatography–mass spectrometry (LC–MS) of intact hopanoid molecules to characterize the extended hopanoids present, a portion of the TLE was analyzed as previously described (Talbot et al. 2003, 2007) using an Agilent 1200 series HPLC and an Agilent 6520 quadrupole time-of-flight mass spectrometer equipped with a Poroshell 120 EC-C18 column (Agilent Technologies) following the protocol of Matys et al. (2019). Hopanoids were identified by retention time and MS2 fragment spectra, and accurate molecular mass and abundances were corrected using authentic standards of diplopterol and BHT kindly supplied by the Summons Lab, Massachusetts Institute of Technology. Since not all compounds shown in Table 2 have authentic standards required for quantification via LC–MS, we do not report relative abundances but simply the presence or absence of a specific compound based on identification of its retention time, MS2 fragment spectra, and accurate molecular mass.

**Table 2:** LC–MS determination of extended hopanoids produced in *Rhodopila* sp. LVNP (structures referenced from Zhu et al. 2011)

Extended Hopanoid	(*m*/*z*)	Structure
3-Me Bacteriohopanetetrol	669	
Aminotriol	714	
Bacteriohopanetetrol	655	
Bacteriohopanetetrol cyclitol ether	1002	
Bacteriohopanepentol cyclitol ether	1060	

Genomic DNA from *Rhodopila* LVNP was isolated using the Genomic Tip 500/G Kit (Qiagen Cat No. 10262) and sequenced by PacBio whole genome sequencing in collaboration with the 2016 Microbial Diversity Course (Marine Biological Laboratory, Woods Hole, U.S.A.). The genome was then assembled and annotated by the Joint Genome Institute annotation pipeline (Huntemann et al. 2015). Auto-annotation of all hopanoid biosynthesis genes was confirmed by manual sequence alignment and visual inspection to confirm the auto-annotation results. This genome is available publicly in the Joint Genome Institute’s IMG Database under the Genome ID Number 2684622831.

The 16S rRNA gene of *Rhodopila* sp. LVNP was amplified using primers 8F and 1492R (Turner et al. 1999) and sequenced in this study; all other sequences were obtained from Genbank and analyzed using BLASTN (Altschul et al. 1997). Nearly complete (~ 1400 nucleotides) 16S rRNA gene sequences were aligned using CLUSTAL (Sievers et al. 2011) and a maximum likelihood phylogenetic tree was generated using PhyML (version 3.2) with the GTR substitution model and bootstrap analysis of 1000 replicates (Guindon et al. 2010). HpnR sequences were identified in the Joint Genome Institute IMG microbial database by protein–protein BLAST (Altschul et al. 1990; Chen et al. 2019), aligned using MUSCLE (Edgar 2004), and maximum likelihood trees built using PhyML (Guindon et al. 2010; Lefort et al. 2017). Our query sequence was the HpnR from *Methylococcus capsulatus* BATH (Locus Name MCA0738; E value 1e-50). The tree was then exported and annotated in iTOL (Letunic and Bork 2019).

## Results

### Characterization of a new *Rhodopila* isolate

Although *Rhodopila* (originally *Rhodopseudomonas*) *globiformis* has been known since 1974, a second isolate of this organism has until now not been reported. In field studies, a spring was discovered in Lassen Volcanic National Park (California, USA) that was geochemically similar to the warm acidic spring that yielded *Rpi. globiformis* (Pfennig 1974) and contained a microbial mat of the strongly acidophilic red alga *Cyanidium* overlying a purple–red layer. Cells from the latter appeared similar to those of *Rps. globiformis* (large weakly motile cocci) and so cultures were pursued and eventually obtained. Considering its habitat, pigments, physiology, and cell morphology, the Lassen purple bacterium was thought to be a new strain of *Rhodopila globiformis* and thus was tentatively designated *Rhodopila* strain LVNP.

**Fig. 2:**
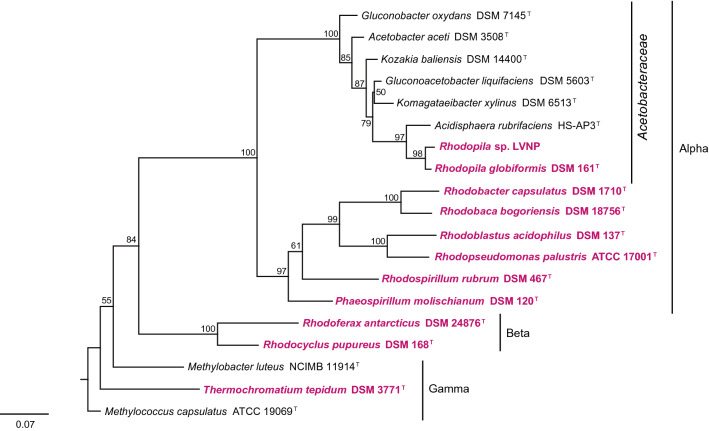
Phylogenetic tree of 16S rRNA genes from some phototrophic purple bacteria and 3-methylhopanoid-producing bacteria. Taxa shown in red are anoxygenic phototrophs and all are PNS bacteria except for the purple sulfur bacterium *Thermochromatium tepidum*. All *Acetobacteraceae* shown and the two methanotrophic bacteria (*Methylobacter luteus* and *Methylococcus capsulatus*) are known producers of 3-methylhopanoids. The labels “Alpha,” “Beta” and “Gamma” refer to classes of the phylum *Proteobacteria*, and numbers at the nodes are bootstrap percentages based on 1000 replications

A 16S rRNA gene phylogenetic tree (Fig. 2) revealed that the Lassen and Yellowstone *Rhodopila* isolates were closely related yet phylogenetically distinct. The percent 16S rRNA gene identity between strain *Rhodopila* sp. LVNP and *Rhodopila globiformis* 7950 YNP (DSM161) was 97%. Moreover, the genome of the Lassen isolate (8.1 Mb) was significantly larger than that of *Rpi. globiformis* (7.2 Mb, Imhoff et al. 2018) and the average nucleotide identity between the two genomes was only 93.1%. Thus, the two strains may be separate *Rhodopila* species rather than strains of the same species. *Rhodopila* is the most acidophilic PNS bacterium (Imhoff and Madigan 2021) and produces unique purple-red carotenoids (Fig. 1c) (Schmidt and Liaaen-Jensen 1973) closely related to okenone, a carotenoid detected in 1.6 Gyr-old rocks from Northern Australia (Brocks et al. 2005; Brocks and Schaeffer 2008). *Rhodopila* is also phylogenetically distinct from other PNS bacteria and is the only anaerobic and acidophilic phototroph that groups with the *Acetobacteraceae*, a bacterial family that includes acetic acid-producing bacteria and other aerobic and acidophilic bacteria (Kersters et al. 2006); this can be seen clearly in Fig. 2.

### Hopanoid analyses

**Fig. 3:**
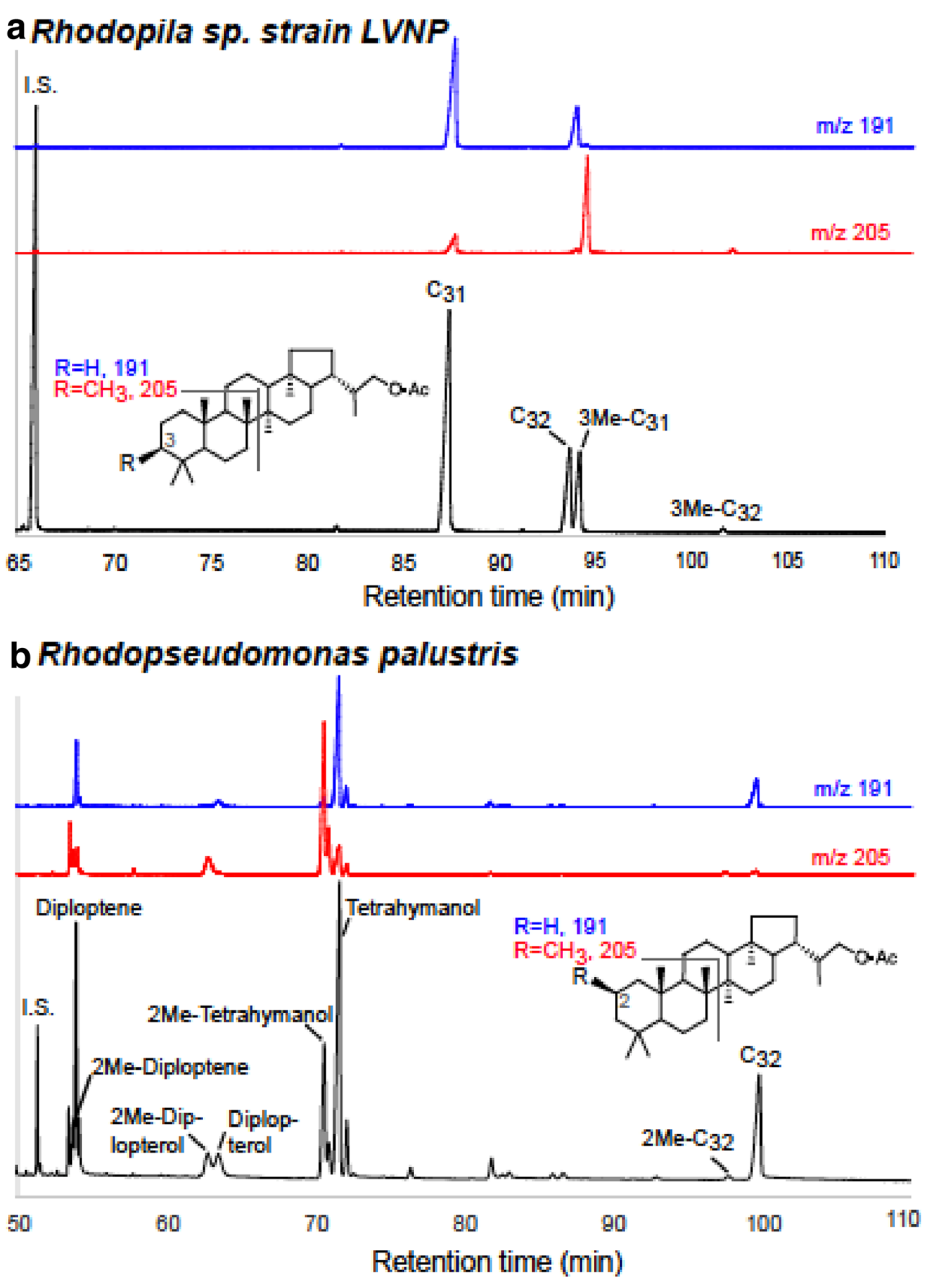
**a** Gas chromatographic–mass spectrometric analyses of 3-methylhopanoids. The compounds, extracted from cultures of *Rhodopila* strain LVNP grown photosynthetically at pH 5, are separated and analyzed as their acetate derivatives. The mass fragment 191 is diagnostic of unmethylated hopanoids (blue chromatogram) while the 205 fragment is diagnostic of their methylated equivalent (red chromatogram). The 3-methylhopanoids elute after their unmethylated equivalents. **b** Analysis of *Rhodopseudomonas palustris* DSM127 as a control. This PNS bacterium produces 2-methylhopanoids (Rashby et al. 2007), which elute before their unmethylated equivalents. I.S., internal standard

Lipid analyses of pure cultures of strain LVNP and the type strain of *Rpi. globiformis* 7950 YNP (DSM 161) grown anaerobically photosynthetically (Pfennig 1974) revealed 3-methylhopanoid production by both strains (Table 1). GC-MSD analyses (Zundel and Rohmer 1985; Summons and Jahnke 1990) confirmed that the hopanoids were indeed 3-methylhopanoids (Fig. 3a) and not 2-methylhopanoids (Fig. 3b). Strain LVNP grown anaerobically photosynthetically at pH 5 synthesized 0.9 µg hopanoid/mg total fatty acid, 0.03 µg 3-methylhopanoid/mg total fatty acid, and 3-methylhopanoids were 14% of the total hopanoid content (3-methylhopanoid/total hopanoid ratio of 0.14). When strain LVNP was grown aerobically in the dark, 3-methylhopanoid production decreased (3-methylhopanoid/total hopanoid ratio of 0.04). The type strain *Rpi. globiformis* 7950 YNP also synthesized less 3-methylhopanoid than the LVNP strain (3-methylhopanoid/total hopanoid ratio of 0.04). Both of these strains synthesized diploptene, but not diplopterol (Table 1). By contrast, phototrophic cultures of the mildly acidophilic PNS bacterium *Rbl. acidophilus* 7050 and the neutrophilic *Rps. palustris* (DSM127) (Fig. 3) did not produce 3-methylhopanoids. Rohmer et al. (1984) also did not detect 3-methylhopanoids in *Rbl. acidophilus* 7050. *Rps. palustris* did, however, contain 2-methylhopanoids as was previously reported (Rashby et al. 2007). Moreover, LC–MS analyses of cells of strain LVNP showed that it produced not only 3-methylhopanoids, but a suite of structurally related hopanoids as well. Although not quantified, LC–MS identified several extended hopanoids, including bacteriohopanetetrols and bacteriohopanetetrol cyclitol ethers (Table 2).

### Genomic evidence for 3-methylhopanoid production in *Rhodopila* species

**Fig. 4:**
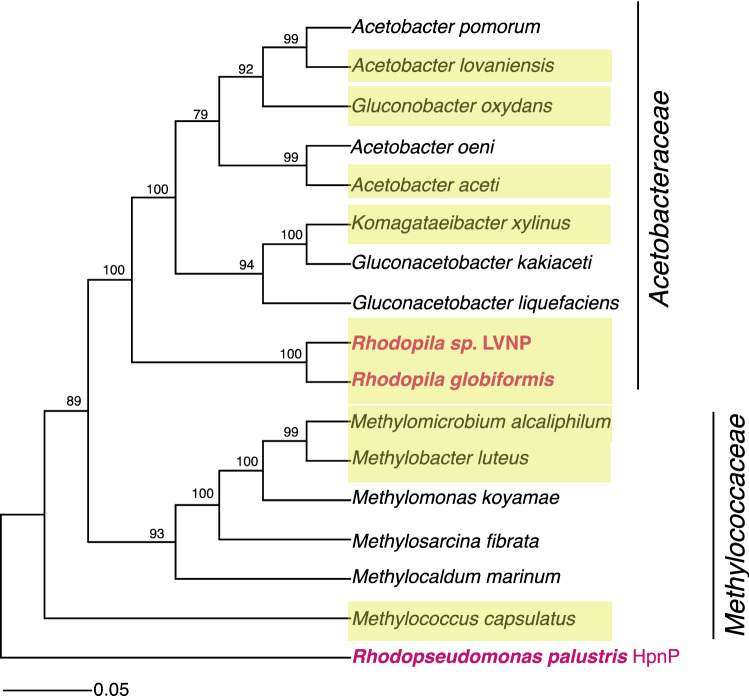
Protein tree of HpnR, the C-3 methylase that methylates hopanoids to form 3-methylhopanoids. Both *Rhodopila* strain LVNP and *Rhodopila globiformis* strain 7950 YNP (DSM161^T^) genomes encode HpnR (Table 3), and the amino acid sequences of this protein cluster with those from their close phylogenetic relatives, the acetic acid bacteria. C-2 hopanoid methylase (HpnP) from *Rhodopseudomonas palustris* was used as the outgroup in the tree. Numbers at the nodes are bootstrap percentages based on 1000 replications. Yellow highlighted taxa denote organisms that have confirmed 3-methylhopanoids in lipids obtained from cultured cells

Genomic analyses in the Joint Genome Institute’s IMG database confirmed that both *Rhodopila* sp. LVNP and *Rpi. globiformis* 7950 YNP (DSM161^T^) (Imhoff et al. 2018) were genetically equipped to produce 3-methylhopanoids (Table 3). Both strains were similar on the basis of their morphology, physiology, and pigments and are also close phylogenetic relatives. The C-3 hopanoid methylase HpnR is encoded in both genomes, and a phylogenetic tree constructed from HpnR sequences (Fig. 4) mirrored the 16S rRNA gene tree (Fig. 2). Specifically, HpnR from the *Rhodopila* species was related to HpnR from species of *Acetobacteraceae* and distinct from that produced by methanotrophic *Methylococcaceae* (Fig. 4); the latter are well-known producers of 3-methylhopanoids but are only distant relatives of *Acetobacteraceae* (Fig. 2). Genes encoding several other hopanoid biosynthesis enzymes (Belin et al. 2018) were identified in the genomes of both *Rhodopila* strains (Table 3) consistent with the production of several related hopanoids identified from cells of *Rhodopila* strain LVNP (Table 2). The gene for tetrahymanol synthase (tsh) was not detected in the *Rpi. globiformis* strains (Query Locus Name MEALZ_1626), as evidenced by the lack of detection of tetrahymanol in lipid extracts (Table 1).

**Table 3:** Locus tags of hopanoid biosynthesis gene homologues in our analyzed cultures of purple nonsulfur bacteria

	Gene function	*Rhodopseudomonas palustris* ATCC 17001 (JGI Genome ID 2516653006)	*Rhodoblastus acidophilus* DSM 137 (JGI Genome ID 2724679731)	*Rhodopila globiformis* DSM 161 (JGI genome ID 2831737867)	*Rhodopila* sp. LVNP (JGI genome ID 2684622831)
*Total genome size (Mb)*	–	5.2	4.7	7.2	8.1
*hpnR*	Methylates at C-3 position	–	–	Ga0347445_1940	Ga0132156_116223
*shc*	Cyclizes squalene into diploptene	RPATCC17001_01169	Ga0170454_103148	Ga0347445_5024	Ga0132156_117315
*hpnC*	Synthesizes the C30 squalene substrate from two isoprenoid precursor farnesyl pyrophosphate (C15) molecules	RPATCC17001_01500	Ga0170454_10337	Ga0347445_3393	Ga0132156_112831
*hpnD*	Synthesizes the C30 squalene substrate from two isoprenoid precursor farnesyl pyrophosphate (C15) molecules	RPATCC17001_01501	Ga0170454_10338	Ga0347445_3392	Ga0132156_112832
*hpnE*	Synthesizes the C30 squalene substrate from two isoprenoid precursor farnesyl pyrophosphate (C15) molecules	RPATCC17001_01170	Ga0170454_10339	Ga0347445_5025	Ga0132156_117314
*hpnH*	First step in side chain development by addition of an adenosyl group to diploptene	RPATCC17001_01164	Ga0170454_10877	Ga0347445_577	Ga0132156_114302
*hpnG*	Cleaves an adenine from the tail end of adenosylhopane for side chain modification	RPATCC17001_01168	Ga0170454_10876	Ga0347445_5023	Ga0132156_117316
*hpnI*	Glycosyltransferase that helps generate the extended hopanoid glucosaminyl BHT	–	Ga0170454_1027	Ga0347445_582	Ga0347445_582
*hpnJ*	Catalyzes a ring contraction to produce a BHT cyclitol ether	–	Ga0170454_101616	Ga0347445_583	Ga0132156_114296
*hpnK*	Deacetylates the BHT acetylglucosamine formed by HpnI to generate the extended hopanoid glucosaminyl BHT	–	–	Ga0347445_584	Ga0132156_114295
*hpnO*	Generates amino BHT	RPATCC17001_01161	Ga0170454_108103	Ga0347445_6048	Ga0132156_112646
*hpnP*	Methylates at C-2 position	RPATCC17001_04569	–	–	–

– indicates gene absent

## Discussion

Our results are the first to show the production of 3-methylhopanoids in bacteria grown anaerobically, thus refuting the contention that these lipids are only produced by obligately aerobic bacteria. It is thus possible, and perhaps even likely, that these lipids are produced by various anaerobes, but only in species of *Bacteria*, since to date no species of *Archaea* have been shown to contain hopanoids (Sahm et al. 1993).

The physiological link between acetic-acid bacteria and *Rhodopila* does not revolve around energy metabolism but instead the ability of both organisms to thrive in strongly acidic habitats. Whether such a lifestyle requires these unusual lipids is unknown, but the fact that 3-methylhopanoids are produced by many neutrophilic methanotrophic bacteria and have not been reported from some other potentially acidophilic bacteria, such as *Thiobacillus* (Rohmer et al. 1979), leaves this question unanswered. Nevertheless, 3-methylhopanoids obviously play some role in the physiology of *Rhodopila* species, and the genetic links between this phototroph and acidophilic bacteria (Fig. 2) and the fact that the analogous 2-methylhopanoids are membrane integrated (Doughty et al. 2009), suggest that 3-methylhopanoids may help maintain membrane function in their acidic habitats. Indeed, a function for hopanoids in maintaining membrane integrity and surviving general environmental stressors has been shown in the cyanobacterium *Nostoc punctiforme* (Ricci et al. 2017). In addition, it has been shown that extended hopanoids aid the chemotrophic bacterium *Bradyrhizobium diazoefficiens* in withstanding hypoxic/low O_2_ growth conditions and various other physiological stressors (Kulkarni et al. 2015). Hence, if there exists a widespread link between 3-methylhopanoids and microbes that inhabit extreme environments, it is possible that in addition to the example we have shown with acidophilic *Rhodopila* species, these hopanoids are produced by anoxygenic phototrophs that thrive in hypersaline, hyperalkaline, and permanently hot or cold environments as well; all of these habitats contain a diversity of purple bacteria (Madigan and Jung 2009; Jahnke et al. 2014).

Production of 3-methylhopanoids by *Rhodopila* highlights the potential importance of anoxygenic phototrophs in the geological rock record and has at least two major geological implications. First, the fact that anaerobically grown *Rhodopila* species can produce 3-methylhopanoids—lipids heretofore observed only in bacteria whose metabolism requires O_2_—indicates that the presence of 3-methylhopanes (the degradation product of 3-methylhopanoids) in ancient rocks can no longer be used as prima facie evidence that oxic conditions existed at the time of deposition. Consequently, linking 3-methylhopanes to O_2_-dependent bacterial metabolisms, such as aerobic methanotrophy (Brocks et al. 2005; Farrimond et al. 2004; Waldbauer et al. 2009), should be done cautiously and only with corroborating evidence.

Second, the relatively high abundance of 3-methylhopanes in mid-Proterozoic marine (Brocks et al. 2005) and Phanerozoic saline lacustrine sediments (French et al. 2020) has been used to infer low sulfate conditions in these aquatic environments, a link that is now called into question. This is because in low sulfate settings, methanogenic *Archaea* typically outcompete sulfate-reducing bacteria for substrates (Hoehler et al. 1998). Thus, in ancient sediments containing 3-methylhopanes, it has been assumed that the increased levels of methane fed the 3-methylhopanoid-containing methanotrophs. However, because our results show that 3-methylhopanes can no longer be unambiguously connected to methanotrophic (or any other obligately aerobic) bacteria, concluding that a given sedimentary rock containing 3-methylhopanes must have formed in low sulfate conditions (Brocks et al. 2005; French et al. 2020) may be erroneous.

## Data Availability

Data from *Rhodopila* strain LVNP are available from MNP upon request. Accession Numbers for 16S rRNA gene sequences obtained from Genbank and used in Fig. 2: *Gluconobacter oxydans* DSM7145 (X73820), *Acetobacter aceti* DSM 3508 (NR_026121), *Kozakia baliensis* DSM 14400 (AB056321), *Gluconoacetobacter liquefaciens* DSM 5603 (X75617), *Komagataeibacter xylinus* DSM 6153 (NR_036787), *Acidisphaera rubrifaciens* HS-AP3 (NR_037119), *Rhodopila* sp. LVNP (MZ461013), *Rhodopila globiformis* DSM 161 (NR_037120), *Rhodobacter capsulatus* DSM 1710 (NR_043407), *Rhodobaca bogoriensis* DSM 18756 (AF248638), *Rhodoblastus acidophilus* DSM 137 (NR_104756), *Rhodopseudomonas palustris* ATCC 17001 (NR_115542), *Rhodospirillum rubrum* DSM 467 (D30778), *Phaeospirillum molischianum* DSM 120 (FR733695), *Rhodoferax antarcticus* DSM 24876 (GU233447), *Rhodocyclus purpureus* DSM 168 (M34132), *Methylobacter luteus* (X72772), *Thermochromatium tepidum* DSM 3771 (MN699348), *Methylococcus capsulatus* ATCC 19069 (NR_042183). IMG Genome IDs for sequences obtained from JGI IMG Database and used in Fig. 3: *Rhodopila* sp. LVNP (2684622831), *Rhodopseudomonas palustris* 7850 DSM 127 (2516653012). IMG Genome IDs for HpnR sequences obtained from JGI IMG Database and used in Fig. 4: *Acetobacter pomorum* (2828617574), *Acetobacter lovaniensis* (2861688124), *Gluconobacter oxydans* (2784746773), *Acetobacter oeni* (2829864053), *Acetobacter aceti* (2784746776), *Komagataeibacter xylinus* (2841172115), *Gluconacetobacter kakiaceti* (2828350867), *Gluconacetobacter liquefaciens* (2756170231), *Rhodopila* sp. LVNP (2684622831), *Rhodopila globiformis* (2831737867), *Methylomicrobium alcaliphilum* (2540341096), *Methylobacter luteus* (2517287033), *Methylomonas koyamae* (2728369704), *Methylosarcina fibrata* (2517487019), *Methylocaldum marinum* (2832923104), *Methylococcus capsulatus* (637000166), *Rhodopseudomonas palustris* (2516653012).

## References

[CR1] Altschul SF, Gish W, Miller W (1990). Basic local alignment search tool. J Mol Biol.

[CR2] Altschul SF, Madden TL, Schäffer AA (1997). Gapped BLAST and PSI-BLAST: a new generation of protein database search programs. Nucleic Acids Res.

[CR3] Belin BJ, Busset N, Giraud E (2018). Hopanoid lipids: from membranes to plant–bacteria interactions. Nat Rev Microbiol.

[CR4] Brocks JJ, Schaeffer P (2008). Okenane, a biomarker for purple sulfur bacteria (Chromatiaceae), and other new carotenoid derivatives from the 1640Ma Barney Creek Formation. Geochim Cosmochim Acta.

[CR5] Brocks JJ, Love GD, Summons RE (2005). Biomarker evidence for green and purple sulphur bacteria in a stratified Palaeoproterozoic sea. Nature.

[CR6] Chen I-MA, Chu K, Palaniappan K (2019). IMG/M vol 5.0: an integrated data management and comparative analysis system for microbial genomes and microbiomes. Nucleic Acids Res.

[CR7] Doughty DM, Hunter RC, Summons RE, Newman DK (2009). 2-Methylhopanoids are maximally produced in akinetes of Nostoc punctiforme: geobiological implications. Geobiology.

[CR8] Edgar RC (2004). MUSCLE: multiple sequence alignment with high accuracy and high throughput. Nucleic Acids Res.

[CR9] Farrimond P, Talbot HM, Watson DF (2004). Methylhopanoids: molecular indicators of ancient bacteria and a petroleum correlation tool. Geochim Cosmochim Acta.

[CR10] French KL, Birdwell JE, Vanden Berg MD (2020). Biomarker similarities between the saline lacustrine Eocene Green River and the Paleoproterozoic Barney Creek Formations. Geochim Cosmochim Acta.

[CR11] Guindon S, Dufayard J-F, Lefort V (2010). New algorithms and methods to estimate maximum-likelihood phylogenies: assessing the performance of PhyML 3.0. Syst Biol.

[CR12] Harwood CS, Gibson J (1988). Anaerobic and aerobic metabolism of diverse aromatic compounds by the photosynthetic bacterium *Rhodopseudomonas palustris*. Appl Environ Microbiol.

[CR13] Hoehler TM, Alperin MJ, Albert DB, Martens CS (1998). Thermodynamic control on hydrogen concentrations in anoxic sediments. Geochim Cosmochim Acta.

[CR14] Hohmann-Marriott MF, Blankenship RE (2011). Evolution of photosynthesis. Ann Rev Plant Biol.

[CR15] Huntemann M, Ivanova NN, Mavromatis K (2015). The standard operating procedure of the DOE-JGI Microbial Genome Annotation Pipeline (MGAP vol 4). Stand Genomic Sci.

[CR16] Imhoff JF, Madigan MT (2021). Rhodopila. Bergey’s manual of systematics of archaea and bacteria.

[CR17] Imhoff JF, Rahn T, Künzel S, Neulinger SC (2018). New insights into the metabolic potential of the phototrophic purple bacterium *Rhodopila globiformis* DSM 161^T^ from its draft genome sequence and evidence for a vanadium-dependent nitrogenase. Arch Microbiol.

[CR18] Jahnke LL, Stan-Lotter H, Kato K, Hochstein LI (1992). Presence of methyl sterol and bacteriohopanepolyol in an outer-membrane preparation from *Methylococcus capsulatus* (Bath). J Gen Microbiol.

[CR19] Jahnke LL, Eder W, Huber R (2001). Signature lipids and stable carbon isotope analyses of octopus spring hyperthermophilic communities compared with those of aquificales representatives. Appl Environ Microbiol.

[CR20] Jahnke LL, Turk-Kubo KA, Parenteau MN (2014). Molecular and lipid biomarker analysis of a gypsum-hosted endoevaporitic microbial community. Geobiology.

[CR21] Kersters K, Lisdiyanti P, Komagata K, Swings J (2006). The Family *Acetobacteraceae*: The Genera *Acetobacter*, *Acidomonas*, *Asaia*, *Gluconacetobacter*, *Gluconobacter*, and *Kozakia*. The Prokaryotes.

[CR22] Kulkarni G, Busset N, Molinaro A (2015). Specific hopanoid classes differentially affect free-living and symbiotic states of *Bradyrhizobium diazoefficiens*. Mbio.

[CR23] Lefort V, Longueville J-E, Gascuel O (2017). SMS: smart model selection in PhyML. Mol Biol Evol.

[CR24] Letunic I, Bork P (2019). Interactive Tree Of Life (iTOL) v4: recent updates and new developments. Nuc Acids Res.

[CR25] Madigan MT, Jung DO (2009). An overview of purple bacteria: systematics, physiology, and habitats. The purple phototrophic bacteria.

[CR26] Matys ED, Mackey T, Grettenberger C (2019). Bacteriohopanepolyols across environmental gradients in Lake Vanda, Antarctica. Geobiology.

[CR28] Pfennig N (1974). *Rhodopseudomonas globiformis*, sp. n., a new species of the Rhodospirillaceae. Arch Microbiol.

[CR29] Rashby SE, Sessions AL, Summons RE, Newman DK (2007). Biosynthesis of 2-methylbacteriohopanepolyols by an anoxygenic phototroph. Proc Natl Acad Sci (USA).

[CR30] Ricci JN, Michel AJ, Newman DK (2015). Phylogenetic analysis of HpnP reveals the origin of 2-methylhopanoid production in Alphaproteobacteria. Geobiology.

[CR31] Ricci JN, Morton R, Kulkarni G (2017). Hopanoids play a role in stress tolerance and nutrient storage in the cyanobacterium *Nostoc punctiforme*. Geobiology.

[CR32] Rohmer M, Bouvier P, Ourisson G (1979). Molecular evolution of biomembranes: structural equivalents and phylogenetic precursors of sterols. Proc Natl Acad Sci (USA).

[CR33] Rohmer M, Bouvier-Nave P, Ourisson G (1984). Distribution of hopanoid triterpenes in prokaryotes. Microbiology.

[CR34] Sahm H, Rohmer M, Bringer-Meyer S et al (1993) Biochemistry and physiology of hopanoids in bacteria. In: Advances in microbial physiology. Academic Press, London, pp 247–273. 10.1016/S0065-2911(08)60100-9 10.1016/s0065-2911(08)60100-9 8310881

[CR35] Schmidt K, Liaaen-Jensen S (1973). Bacterial carotenoids. XLII. New keto-carotenoids from *Rhodopseudomonas globiformis* (Rhodospirillaceae). Acta Chem Scand.

[CR36] Sessions AL, Zhang L, Welander PV (2013). Identification and quantification of polyfunctionalized hopanoids by high temperature gas chromatography–mass spectrometry. Org Geochem.

[CR37] Sievers F, Wilm A, Dineen D (2011). Fast, scalable generation of high-quality protein multiple sequence alignments using Clustal Omega. Mol Syst Biol.

[CR38] Summons RE, Jahnke LL (1990). Identification of the methylhopanes in sediments and petroleum. Geochim Cosmochim Acta.

[CR39] Summons RE, Jahnke LL, Hope JM, Logan GA (1999). 2-Methylhopanoids as biomarkers for cyanobacterial oxygenic photosynthesis. Nature.

[CR40] Talbot HM, Squier AH, Keely BJ, Farrimond P (2003). Atmospheric pressure chemical ionisation reversed-phase liquid chromatography/ion trap mass spectrometry of intact bacteriohopanepolyols. Rapid Commun Mass Spectrom.

[CR41] Talbot HM, Rohmer M, Farrimond P (2007). Rapid structural elucidation of composite bacterial hopanoids by atmospheric pressure chemical ionisation liquid chromatography/ion trap mass spectrometry. Rapid Commun Mass Spectrom.

[CR42] Turner S, Pryer KM, Miao VPW, Palmer JD (1999). Investigating deep phylogenetic relationships among cyanobacteria and plastids by small subunit rRNA sequence analysis. J Eukaryot Microbiol.

[CR43] Waldbauer JR, Sherman LS, Sumner DY, Summons RE (2009). Late Archean molecular fossils from the Transvaal Supergroup record the antiquity of microbial diversity and aerobiosis. Precambr Res.

[CR44] Welander PV, Summons RE (2012). Discovery, taxonomic distribution, and phenotypic characterization of a gene required for 3-methylhopanoid production. Proc Natl Acad Sci (USA).

[CR45] Yildiz FH, Gest H, Bauer CE (1991). Attenuated effect of oxygen on photopigment synthesis in Rhodospirillum centenum. J Bacteriol.

[CR46] Zhu C, Talbot HM, Wagner T (2011). Distribution of hopanoids along a land to sea transect: Implications for microbial ecology and the use of hopanoids in environmental studies. Limnol Oceanogr.

[CR47] Zundel M, Rohmer M (1985). Prokaryotic triterpenoids. Eur J Biochem.

